# Blue Laser for Production of Carbon Dots

**DOI:** 10.3390/polym16192801

**Published:** 2024-10-03

**Authors:** Mariapompea Cutroneo, Petr Malinsky, Petr Slepicka, Lorenzo Torrisi

**Affiliations:** 1 Dipartimento di Scienze Matematiche e Informatiche, Scienze Fisiche e Scienze della Terra, MIFT, Università di Messina, V.le F. Stagno d’Alcontres 31, 98166 Messina, Italy; 2 Nuclear Physics Institute, AS CR, 250 68 Rez, Czech Republic; malinsky@ujf.cas.cz; 3 Department of Physics, Faculty of Science, University of J. E. Purkyně, České mládeže 8, 400 96 Ústí nad Labem, Czech Republic; 4 Department of Solid State Engineering, University of Chemistry and Technology Prague, 166 28 Prague, Czech Republic

**Keywords:** carbon dots, luminescence, PBS, laser ablation in liquid, polycaprolactone

## Abstract

The synthesis of carbon dots (CDs) is gaining wide-ranging interest due to their broad applicability, owing to their small size and luminescence. CDs were prepared from charcoal via a one-step process using laser ablation in liquid without the use of reagents. The adopted method was based on the use of a commercially available continuous wave (CW) laser diode emitting a 450 nm wavelength and, for the liquid, a phosphate-buffered saline (PBS) solution, routinely used in the biological field. Photoluminescence analysis revealed fluorescence, at 480 nm, increasing with laser irradiation time. The atomic force microscopy (AFM) of the CDs revealed an average sphere shape with a size of about 10 nm. Biodegradable polycaprolactone (PCL), typically adopted in biomedicine applications, was used as a matrix to show the preserved luminescence, ideal for the non-invasive monitoring of implanted scaffolds in tissue engineering.

## 1. Introduction

Carbon dots (CDs) are fluorescent carbon nanoparticles with diameters below 10 nm. Since their first appearance in 2004 [1], the amorphous variety of these quasi zero-dimensional structures have gathered less attention with respect to crystalline structures such as fullerene, graphene quantum dots, and carbon quantum dots, to mention a few [2].

The benefits offered by amorphous CDs include their easy synthesis, water solubility, biocompatibility, and nontoxicity. The potential environmental hazard they create, together with their toxicity, may represent serious limitations to the use of nanoparticles both in vitro and in vivo [3,4].

Studies such as that of Cao et al. [5] reported that amorphous CDs have properties comparable to that of crystalline CDs [6]. Up to now, the photoluminescence mechanism of crystalline and amorphous CDs has still not been recognized as the main issue of the luminescence properties in the source.

On the contrary, there is a general consensus regarding the involvement of surface traps in the radiative transition of carbon dots. It seems that the surface groups of C-O, C=O, and O=C-OH can induce trapping states with different energy levels. This results in the emission of CDs at different excitation energies [7]. An interesting study performed by Yu et al. [8] related the dependence of CDs luminescence to temperature.

In the scenario of luminescence induced by amino-functionalized CDs, the peaks at 420–460 nm are ascribed to n-π* transitions and to the doping of CDs with oxygen and nitrogen heteroatoms [9].

Approaches for the production of CDs range from electrochemical oxidation [10] and microwave synthesis [11] to organic carbonization [12].

Most studies performed on CDs with significant photoluminescent properties have used bottom-up techniques based on chemical routes, such as surface passivation, synthesis from candle soot, or the hydrothermal carbonization of citric acid [13]. It is worth remembering that these approaches usually involve complex processes and extreme synthesis conditions, and result in frequently toxic byproducts.

However, the recent literature mentions the use of lasers as a promising method for the synthesis of CDs at moderate costs, without the need for vacuum systems, and using a set up that is easily implemented. Lasers can be used in fundamental research [14], material modification [15], and the production of nanoparticles [16].

The proper operation of the technique, known as pulsed laser ablation (PLA), in liquid is based on the control of the laser irradiation parameters, like irradiation time, repetition rate, pulse duration, and wavelength, and of the liquid’s features, like the pH and the dielectric constant [17].

PLA has been routinely used to produce metallic nanoparticles from Auto Cu, and even semiconductors like ZnO [18] or Bi. It seems that carbon nanoparticles produced by laser ablation in water display negligible fluorescence, but when using other liquids like acetone, isopropyl alcohol, polyethylene glycol, ethylenediamine, or polyethyleneimineare, the nanoparticles reveal their photoluminescence [2,3,4,5,6,7,8,9,10,11,12,13,14,15,16,17,18,19,20].

Despite other works reporting on the production of CDs using urea as a reagent [2], in this proposed study, no reagents resulting in luminescent properties, like the hazardous amine-based reagents ethylenediamine (EDA) and polyethyleneimine (PEI), were employed. Recently, CDs have been embedded in polymers for the production of functionalized bulk, membranes, and sensors.

Polycaprolactone (PCL) is an emerging hydrophobic semi-crystalline polymer, widely appreciated for its biodegradability and biocompatibility [21].

PCL can be used in tissue engineering, as approved by the United States Food and Drug Administration [22].

Its mechanical properties, flexibility, and thermal stability, along with the antibacterial, antioxidant, and UV barrier properties of CDs, make its combination with CDs an alternative method of enhancing the shelf life of edible products [23]. Ezati et al. reported on the antimicrobial and antioxidant behavior of packaging material filled with CDs for the conservation of food [24,25]. The benefits of the use of polycaprolactone with respect other polymers like poly(lactic-co-glycolic acid) include its slow degradation rate, its easy manufacturing, and its mechanical qualities [26]. The development of functional fluorescent polycaprolactone (PCL) could be crucial in tissue engineering for offering a proper contrast between the fluorescence emitted by cells from that of PCL scaffolds [27,28].

Table 1 displays brief summaries of the advantages and disadvantages offered by the most used technique for the production of CDs. The process used in our study was PLA. It is considered a complex process requiring reactants and subsequent purification, depending on the application of the produced CDs. Here, the laser assisted the production of biocompatible CDs in a biocompatible environment without the use of activators, without the need for any purification, and definitely using a very affordable source: common charcoal. Moreover, during PLA, laser pulses with high repetition rates induce cavitation bubbles responsible for the shielding of the successive laser pulses, the consequent loss of the effective laser energy reaching the target, and the reduction in CD productivity [29]. Presently, the use of a continuous laser instead of a pulsed-periodic one has been proposed for the first time by our group [30,31], as far as we know, and could lead to the no-cost intensive productivity of CDs.

This work is focused on the preparation of CDs by laser ablation in phosphate-buffered saline (PBS) solution using a commercially available diode laser. We additionally report on morphological and optical analyses of produced CDs, and then we highlight the possible use of polycaprolactone (PCL) impregnated with synthetized CDs.

The biocompatible CDs produced without the use of harmful or toxic reagents and embedded in polycaprolactone (PCL) tissue could be applied in tissue engineering due to their green composition and optical features.

## 2. Material and Methods

We used a semiconductor GaAs laser, which is typically labeled a blue laser as it emits coherent light at a wavelength of 450 nm, a bandwidth of 200 kHz full width at half maximum (FWHM), an outpower of 50 W, and an intensity of 5 × 10^3^ W/s cm^2^. The laser, equipped with an inner focus system enabling an output beam size between 1 cm and 1 mm, was used in the continuous wave mode (CW) and at a 1 mm^2^ spot size. Phosphate-buffered saline (PBS) was purchased from Sigma Aldrich (Darmstadt, Germany). One tablet with a weight of 2 g was dissolved in 200 mL of deionized water [35] and immediately used as an environment for pulsed lased ablation in liquid to preserve its purity. A piece of charcoal originating from tall trees in eastern Sicily (Italy) was used as a source for the production of CDs. It exhibited a density of about 0.57 g/cm^3^ [36] and a porosity of about 30%. The average wt % composition for the charcoal reported in the literature is as follows [37,38]: C = 66.9%: H = 4.4%; O = 7.6%; N = 1.3%; S = 1.1%; Moisture = 7.2%; Ash = 11.5%; Cl = 0.1%. However, its composition may be altered with changes in temperature, burning time, oxygen content, type of burned wood, and geographic area. Charcoal carbon embodies many functional oxygen groups, from carbonyl groups (C=O), carboxyl groups (O=C-OH), and epoxide groups (-C-O-C-) to hydroxyl groups (-OH), and H_2_O, CO_2_, N_2_, and C-N, among other gasses.

### 2.1. Process of Laser Ablation in Liquid

The laser irradiating the surface of the target immersed in liquid is absorbed by the target.

The interface between the solid surface and the liquid is heated to temperatures in the order of several kilokelvins, leading to the formation of plasma, confined by the liquid. During the expansion and condensation of the plasma plume, a vapor layer is formed. The expansion of the vapor layer forms a cavitation bubble, and the plasma shrinks with the compression of the vapor. The pressure can reach several GPa, which forces chemical interaction between the vaporized medium and the ablated species [39]. 

The new solid phase structures formed inside the plasma and at the plasma–liquid interface are dispersed in the liquid when the bubble is broken. The obtained structures grow until they reach the liquid temperature [40].

**Figure 1:**
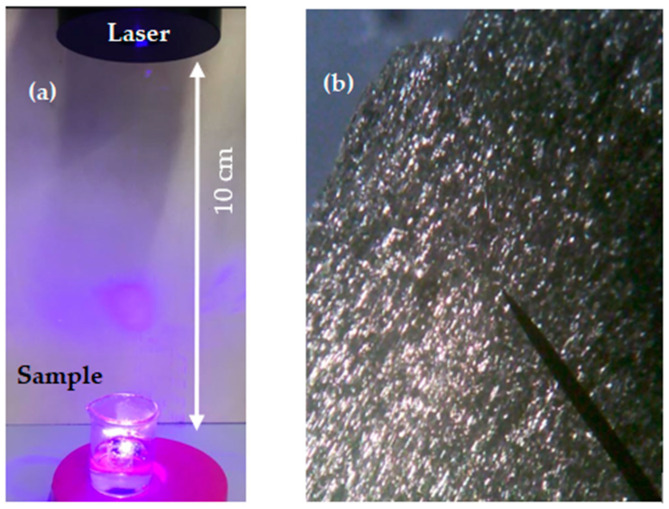
Image of the PLA set up (**a**) and optical image of the charcoal target (**b**).

The used charcoal had an irregular shape 1.8 × 1.4 cm and is 5 mm in height. It was located in a glass vessel containing 4 mL of PBS at a controlled pH of 7. Figure 1 shows the set up adopted for the preparation of the CDs (see Figure 1a) and the optical image of the used charcoal target magnified by x4 (see Figure 1b). The laser beam size was 1 mm^2^ and the target was manually rotated every 5 min.

The target was covered by 1 mm of liquid and laser irradiated for 30 and 60 min.

### 2.2. Characterization of the Produced CDs and the PCL + CDs Composites

The PCL + CDs composites were studied using attenuated total reflectance coupled with Fourier transform infrared spectroscopy (ATR-FTIR) and were monitored using a JASCO Model 4600 spectrophotometer working in the (400–4000) cm^−1^ wavenumber range.

The luminescence of the produced CDs was observed using the Avantes AvaSpec-2048-USB2 optical spectrometer. The luminescence was monitored in the transmission mode in the region 200–800 nm. The exciting UV light source operating at 365 nm and at a fluence of about 100 mJ/cm^2^ illuminated the front of the cuvette containing the CD suspension at a 10 cm distance and at 0°, while the fiber connected to the spectrometer was located on the back of the cuvette at a 1 mm distance and at 180°.

The silicon cuts 1 cm × 1 cm in size were covered with drops of CD suspension and dried in air overnight. The formed films were studied by AFM. A dimension ICON AFM system (Bruker Corp., Bremen, Germany) operating in the ScanAsyst imaging mode in air has been employed. A commercial silicon Tip and SCANASYST, in air mode, with a spring constant of 0.4 N/m, supported 3 μm^2^ scanning. The identification of the CDs was carried out using AFM images recorded and processed using NanoScope Analysis 1.80 with 32-bit software.

## 3. Results and Discussion

**Figure 2:**
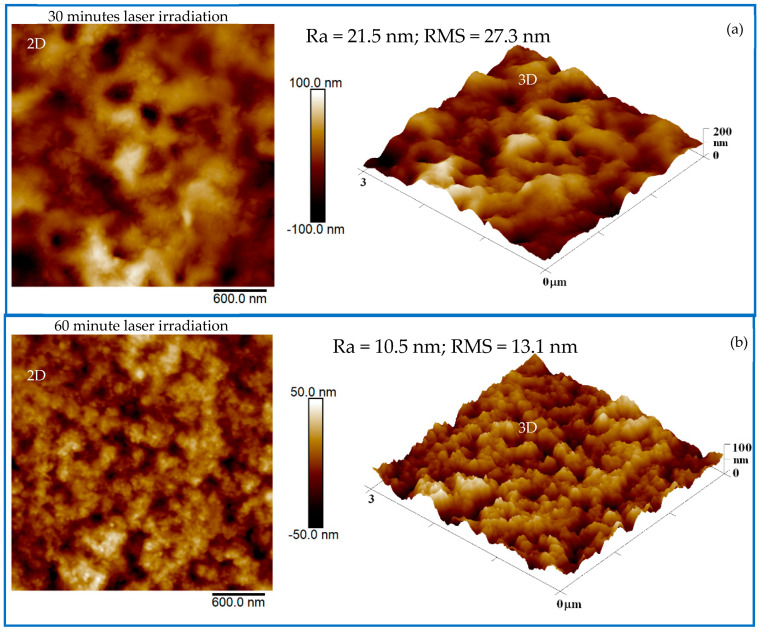
**Two-dimensional** and three-dimensional AFM images of PBS+ CDs 30 min (**a**) and PBS + CDs 60 min (**b**).

The presented results are divided into two main parts. The first part focuses on the description of the adopted route and the preliminary characterization of the CD suspensions produced by PLA. The last part describes the possible use of PCL containing CDs. The suspensions obtained by laser ablation in liquid were poured on cuts of silicon and dried in air overnight. The 3 μm^2^ scanning of AFM images performed on these cuts is shown in Figure 2.

In Figure 2, the 3D images assist in the better recognition of the structures formed during the laser ablation of charcoal in liquid. Figure 2a shows circular structures with sizes of about 21 nm assigned to the PBD + CDs 30 min. Figure 2b shows better-defined circular structures with sizes of about 10 nm.

**Figure 3:**
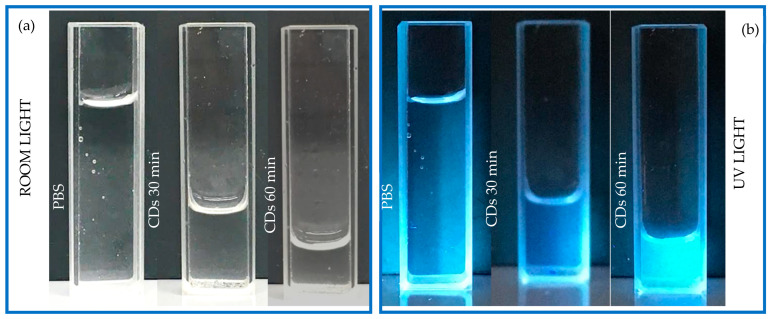
Photos of the vessels containing PBS, PBS+ CDs 30 min, and PBS+ CDs 60 min illuminated by room light (**a**) and by UV light (**b**).

In accordance with AFM analysis, one assumes an average spherical shape of the CDs with a diameter of 10 nm. This work is in progress to further investigate the size and nature of CDs. The material removed for one hour of charcoal laser irradiation was of about 0.017 g and had an assumed charcoal density of 0.57 g/cm^3^ and an average volume of the nanoparticles (NPs) of about 523 nm^3^. It is reasonable to assume a production of 5.7 × 10^16^ NP/cm^3^ was achieved. Figure 3 shows the qualitative luminescence of pristine PBS solution compared to the suspensions of CDs obtained after 30 min and 60 min of laser irradiation. Figure 3a displays the suspensions in quartz cuvettes illuminated by room light, while Figure 3b reveals the luminescence of the same suspensions illuminated by UV light.

All the suspensions appear transparent under the white visible light (see Figure 3a) and blue-shaded under the irradiation of the UV light (see Figure 3b). The suspension CDs + 30 min discloses a very poor luminosity, close to that of PBS. On the contrary, the suspension CDs + 60 min exhibits better luminescence.

**Figure 4:**
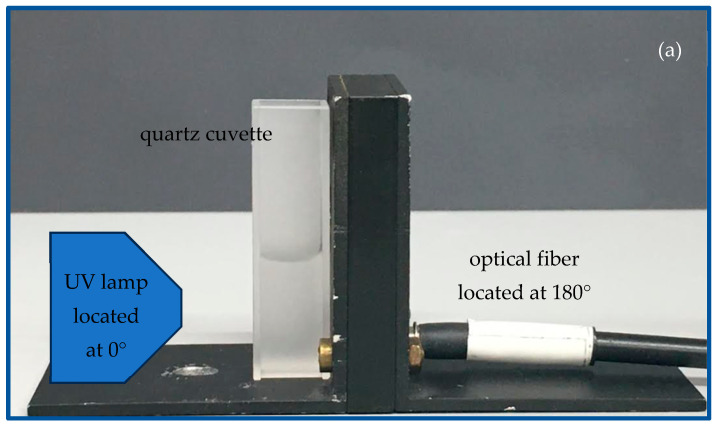

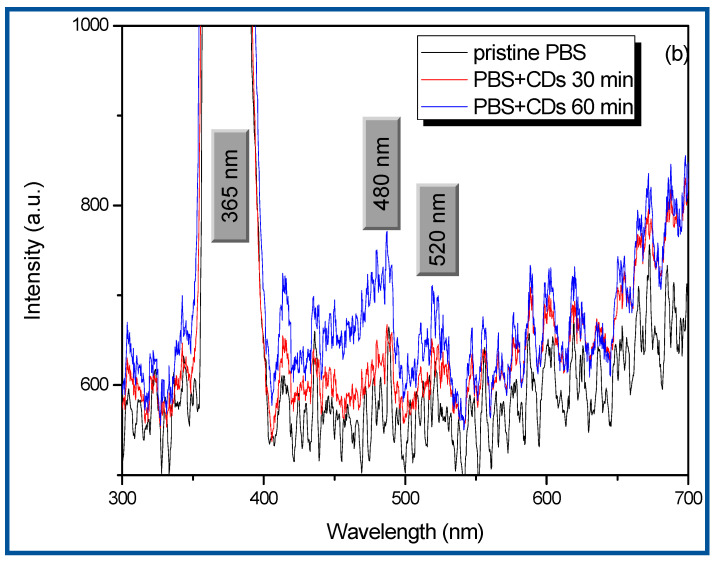
Set up for CD luminescence induced by UV lamp (**a**) and luminescence spectrum (**b**).

To validate the empirical luminescence of the CDs + 60 min suspensions, optical spectroscopy was carried out using the system presented in Figure 4a.

The first peak in Figure 4b shows the excitingUV source centered at a 365 nm wavelength. The visible luminescence of the suspensions is marked out by the peaks at 480 nm and 520 nm (see Figure 4b) through a fiber, 300 nm in diameter, and positioned at 0° in front of the cuvette (see Figure 4a).

The luminescence of the suspensions seems to be affected by the concentration of the formed CDs, which is related to the irradiation time of the charcoal during PLA. The peak at 480 nm increases by about 0.94% after 30 min and by about 17.2% after 60 min. The peak at 520 increases by about 6.1% after 30 min and by about 15.8% after 60 min.

The energy per photon for light exciting the CDs’ luminescence, with a wavelength of 365 nm, is of 3.39 eV, while the energy of the photons emitted by the luminescence at 520 nm is 2.38 eV, and at 480 nm is 2.56 eV.

As a result, the photons emitted from the UV lamp are absorbed by molecular electrons in the valence band. These electrons acquire enough energy to pass into the conduction band, and then after less than 10^−12^ s, spontaneously decay in the valence band, or in intermediate levels above the valence band, and are revealed through a characteristic luminescent emission of light [35].

Typically, CD luminescence can be induced in the following ways: altering the types or contents of surface functional groups (the surface state); electron transitions of the conjugated sp^2^ domains, indicated as the carbon core state; or through the molecular fluorophores or their aggregates, referred to as the number of emitted photons per absorbed photon (the molecular state) [41].

The prepared suspensions containing CDs have been used to immerse pieces of tissue of polycaprolactone (PCL), which are then dried in air at room temperature for 24 h.

**Figure 5:**
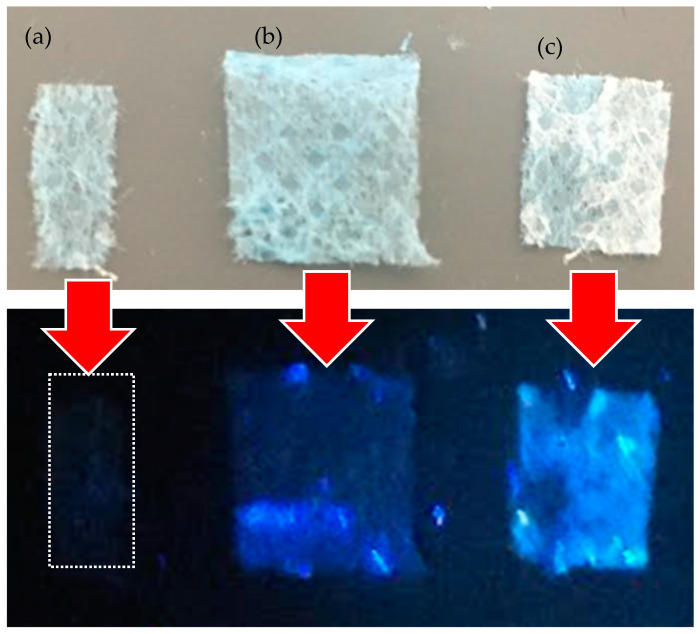
Images of PCL cuts dried (**a**), wet with CDs 30 min (**b**), wet with CDs for 60 min (**c**), and illuminated by visible and UV light, respectively.

In Figure 5, the dried PCL is barely visible under UV, while the PCL cuts soaked in CDs for 30 min (see Figure 5b) and the CDs for 60 min (see Figure 5c) show an increasing luminescence.

**Figure 6:**
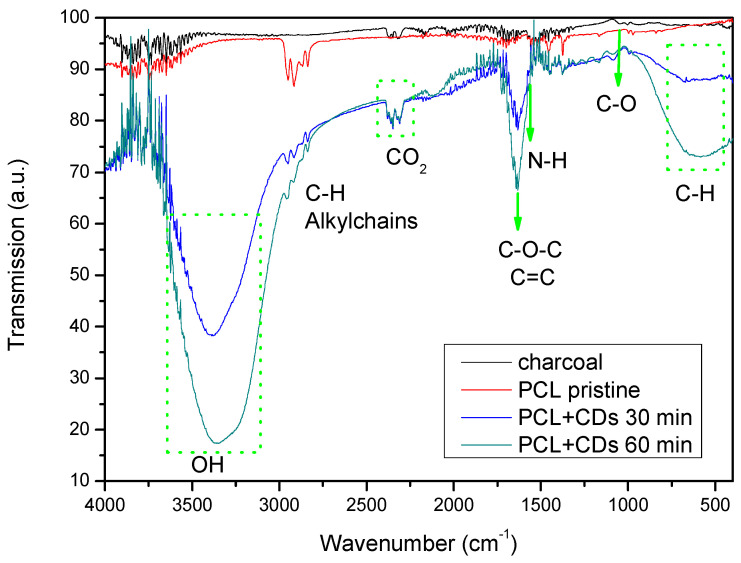
ATR-FTIR spectra of the PCL cuts soaked in CDs for 30 min and in CDs for 60 min, respectively.

To investigate the presence of both PCL and CDs, Fourier transform infra-red (FTIR) spectroscopy was performed. The spectra obtained analyzing the polycaprolactone impregnated with CD suspensions and then gently dried, are reported in Figure 6.

The IR spectrum of pristine PCL seems to be in good agreement with the polycaprolactone structure [42].

The spectrum of the PCL matrix shows peaks between 2972 and 2826 cm^−1^ corresponding to alkylchains, and a very sharp signal at 1750 cm^−1^, corresponding to ester groups [43].

The peaks marked by green arrows are characteristic of CDs. At 1098 cm^−1^, the stretching vibration of C–O is revealed, while at 1637 cm^−1^, the vibration of the C-O-C bond is revealed. The broad absorption band displayed in the range of 580–780 cm^−1^ is assigned to the stretching vibration of C-H in methylene [44]. The absorption band is assigned to the bending vibration of N-H at 1570 cm^−1^. The stretching vibrations of C=C and/or C=N (~1600–1610 cm^−1^) bonds suggest the presence of carbon aggregation or CDs [45].

Different oxygen functional groups are present in the cuts of PCL impregnated with CDs. A broad peak between 3644 cm^−1^ and 3000 cm^−1^ assigned to the hydroxyl (-OH) is clearly visible [46].

The biocompatibility of both the produced CDs and the PCL, besides their low immunogenicity and low degradability, make this a promising composite for scaffold preparation [47] and biological studies [48]. The adopted route for the manufacturing of PCL tissue containing CDs offers a number of advantages, like not requiring tricky and long production steps, nor the use of any other material for the incorporation of CDs, and not requiring nitrogen and sulfur doping for the improvement of their physical and chemical characteristics [49]. The PCL containing CDs fulfills another feature crucial for the efficiency of a scaffold assisting integration to the host tissue and the regeneration of the damaged tissue: the presence of a spatial environment for cell growth and a physiochemical substrate for cell attachment. The cuts of the PCL tissue impregnated with the produced luminescence suspension containing CDs could be used to improve the growth of cell cultures in tailored patterns, as well as to assist in the fluorescent staining of cells on scaffolds.

## 4. Conclusions

In this study, we produced CDs in biocompatible liquid without the use of activators and reagents that could make the final product toxic, as reported in the literature. We adopted pulsed laser ablation in PBS liquid, and we used as a primary target a commonly available charcoal. The luminescence of the fabricated CDs was proved by irradiating the fabricated suspensions under UV light and recording the emitted light at 480 nm and 520 nm, corresponding to blue light. The average size of the CDs was estimated to be less than 10 nm by AFM analysis. Cuts of polycaprolactone tissue were impregnated with the suspensions obtained after 30 min and 60 min of laser ablation. The CDs were anchored inside and on the surface of the tissue, as revealed by FTIR analysis. The designed cuts of PCL containing CDs could be a promising alternative to current bone scaffolds. The advantage of this in implant bone grafts could be a superior assisted bone regeneration. The natural porosity of the PCL tissue could promote cell adhesion and better cell distribution. In consideration of the biocompatibility of the CDs and PCL, the tissue impregned with CDs could lead to more favorable cell survival, while the tunable fluorescence emission could lead to the manufacturing of wearable, low-cost, and efficient sensing devices for environmental monitoring and health care.

Work is in progress to investigate in more depth the size of the produced CDs and the possibility of using the biocompatible luminescent solutions to promote a better recognition of fluorescent cell cultures on scaffolds.

## Figures and Tables

**Table 1:** Comparison between different methods of manufacturing CDs.

	Advantage	Disadvantage
Chemical method [32]	It avoids the use of both expensive beginning materials and energy systems.	The use of strong acids such as nitric acid in large quantities.
Carbonization of molecular precursors such as glucose, sucrose, glycerol, citric acid, ascorbic acid, and biowaste	It produces fluorescent CQDs.	CDs obtained from natural sources using chemicals for modification can lead to CD toxicity; this is a multi-step process.
Hydrothermal method [33]	It is performed in a single environment and it is economically advantageous.	It requires expensive autoclaves and involves safety issues during the reaction process.
Laser ablation technique	It is very fast and easily implemented.	It is not economically advantageous, it is complex, and it requires the use of reactants and a subsequent purification process [34].

## Data Availability

The raw data supporting the conclusions of this article will be made available by the authors on request.
